# Discovery of a Functional Sequon for Chondroitin Sulfate Glycosylation

**DOI:** 10.21203/rs.3.rs-10307688/v1

**Published:** 2026-07-16

**Authors:** Yoshiko Takeda-Uchimura, Mayumi Ishihara-Aoki, Ayano Moriya, Kazuchika Nishitsuji, Shuji Mizumoto, Midori Ikezaki, Yuki Takechi-Haraya, Eriko Nakato, Hassan Lemjabbar-Alaoui, Fabrice Allain, Yoshito Ihara, Shuhei Yamada, Michael Tiemeyer, Hiroshi Nakato, Kazuhiro Aoki, Kenji Uchimura

**Affiliations:** 1 Univ. Lille, CNRS, UMR 8576-UGSF- Unité de Glycobiologie Structurale et Fonctionnelle; F-59000 Lille, France.; 2 Complex Carbohydrate Research Center, University of Georgia; Athens, GA 30602 USA.; 3 Mass Spectrometry Core, Translational Metabolomics Shared Resource, Cancer Center, Medical College of Wisconsin; Milwaukee, WI 53226, USA.; 4 Department of Genetics, Cell Biology, and Development, University of Minnesota, Minneapolis; MN 55455, USA.; 5 Department of Biochemistry, Wakayama Medical University; Wakayama, 641-8509, Japan.; 6 Department of Pharmacy, Meijo University; Nagoya, 468-8503, Japan.; 7 Division of Biochemistry, National Institute of Health Sciences; Kawasaki, 210-9501, Japan,; 8 Department of Surgery, University of California, San Francisco; CA, 94143, USA.; 9 Department of Cell Biology, Neurobiology and Anatomy, Medical College of Wisconsin; Milwaukee, WI 53226, USA.

## Abstract

The amino acid sequence requirements that instruct the modification of proteins with chondroitin sulfate (CS) have been unknown, precluding predictions or opportunities for precise protein engineering. This study identified an essential amino acid motif for CS addition, the “CS-sequon” (EDQDDKDGGDFSGWGG), by comparing the secreted sulfatases SULF1 and SULF2, where only SULF2 is CS-modified. A cluster of seven amino acids plus a nearby tryptophan are critical for CS attachment; inserting the CS-sequon into SULF1 enabled it to gain CS modification. This sequon recruits the activity of the CS-initiating xylosyltransferase to the peptide modification site and CS addition enhances SULF1/2 extracellular abundance and enzymatic activity. These findings were validated across human and non-human cells and in Drosophila, providing a foundational resource for engineering CS modifications into proteins.

SULF1 and SULF2 are extracellular endoglucosamine-6-sulfatases previously identified in humans^1^. The SULFs regulate the sulfation patterns of heparan sulfate proteoglycans (HSPGs), thereby modulating multiple signaling pathways, including those of morphogens, growth factors, and cytokines. SULF protein activities play important roles in development, tissue homeostasis, and disease processes. Although they share high amino acid sequence similarity and enzymatic function, previous studies have demonstrated a striking difference in the post-translational modification of the SULFs. SULF2 is glycosylated by the addition of chondroitin sulfate (CS), while SULF1 is not^2^. The molecular mechanisms underlying this difference remain unknown. It is also uncertain how the presence or absence of CS glycosylation impacts the localization and function of the SULF proteins.

Glycosylation is a major post-translational modification found on many proteins. Certain glycosylations are dictated by the amino acid sequence of the modified proteins. A “sequon” refers to a specific sequence of consecutive amino acids within a protein that functions as a site for the attachment of a glycan. This concept is well established for N-linked glycosylation, where the linkage between the N-glycan and the protein occurs through asparagine (Asn). The canonical sequon for N-glycosylation is characterized by the pattern Asn-X-serine (Ser) or Asn-X-threonine (Thr)^3, 4, 5^. CS is a member of the glycosaminoglycan (GAG) family, polysaccharides that are frequently sulfated and are post-translationally attached to proteins. Heparan sulfate (HS) is another member of this family. Both CS and HS are initiated by the addition of a structure called a xylosylated tetrasaccharide bridge, in which xylose is covalently linked to the Ser residues of the protein, and then two galactose (Gal) residues and a glucuronic acid (GlcA) are linked. An amino monosaccharide, either N-acetylgalactosamine (GalNAc for CS) or N-acetylglucosamine (GlcNAc for HS), is attached. The tetrasaccharide bridge serves as the substrate for subsequent elongation with a repeating disaccharide that is characteristic of either CS (-GalNAc-GlcA-) or HS (-GlcNAc-GlcA-). These GAG-glycosylated Ser residues are often found in Ser-glycine (Gly) sequences^6^ and are sometimes associated with proximal acidic amino acids^7, 8^. However, the primary and secondary structures, along with the essential sequence elements near the Ser residue that determine the addition and elongation of CS glycans, remain unclear.

We sought to identify the flanking amino acid sequences that regulate CS modification by exploiting the difference between SULF2 and SULF1. We hypothesized that a functional sequon for CS addition exists in SULF2 but not in SULF1, and that introduction of this sequon would be sufficient to create a CS-modified form of SULF1. Here, we demonstrate that a cluster of seven consecutive amino acid residues, including five acidic and one basic residue in close proximity to the Ser undergoing CS addition, is essential for modification in SULF2. We also found that the introduction of the aromatic amino acid tryptophan (Trp) two residues downstream from the Ser dramatically increases the proportion of SULF2 undergoing CS modification. Interestingly, introducing three consecutive Ser-Gly sequences adjacent to this Ser increased the length of the attached CS glycan. These results were reproducible in both human HEK293 cells and non-human CHO cells. Notably, insertion of our identified CS sequon, EDQDDKDGGDFSGWGG, into SULF1 resulted in a novel CS-modified form of the protein. These regulatory sequences do not alter the secondary structure of the peptides, but rather control the substrate specificity of the GAG-initiating xylosyltransferase (XYLT)^9, 10, 11, 12^, the first enzyme involved in synthesis of the tetrasaccharide bridge. These findings were further confirmed for Drosophila SULF proteins in vitro and in vivo and demonstrated that the addition of CS increased the abundance of the SULF proteins in the extracellular space and enhanced their endoglucosamine-6-sulfatase activities. The CS sequon described here provides a strategy to biosynthetically introduce CS modifications into extracellular proteins and to further explore the biological roles of CS-modified proteins.

## Results and Discussion:

### Identification of the acidic amino acid cluster required for CS modification in SULF2 and its enhancement by tryptophan substitution

**Fig. 1.**
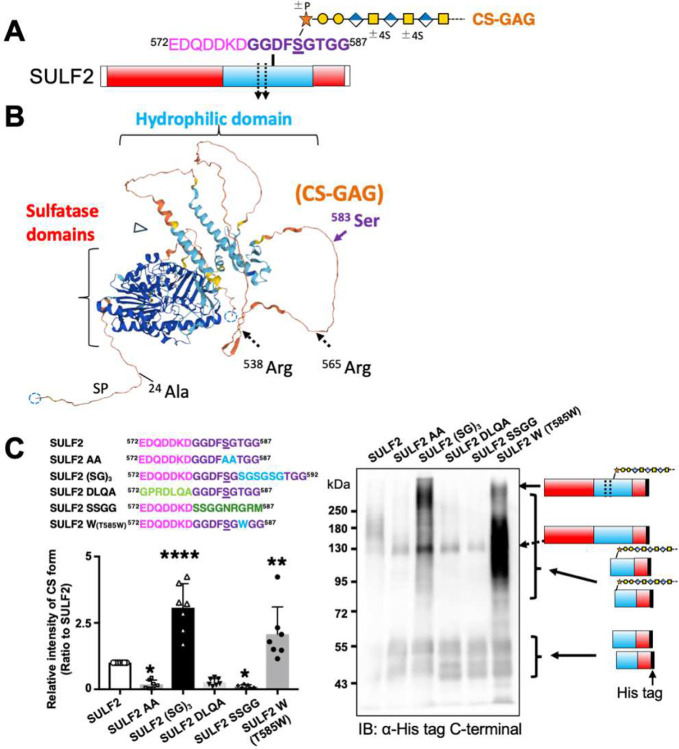

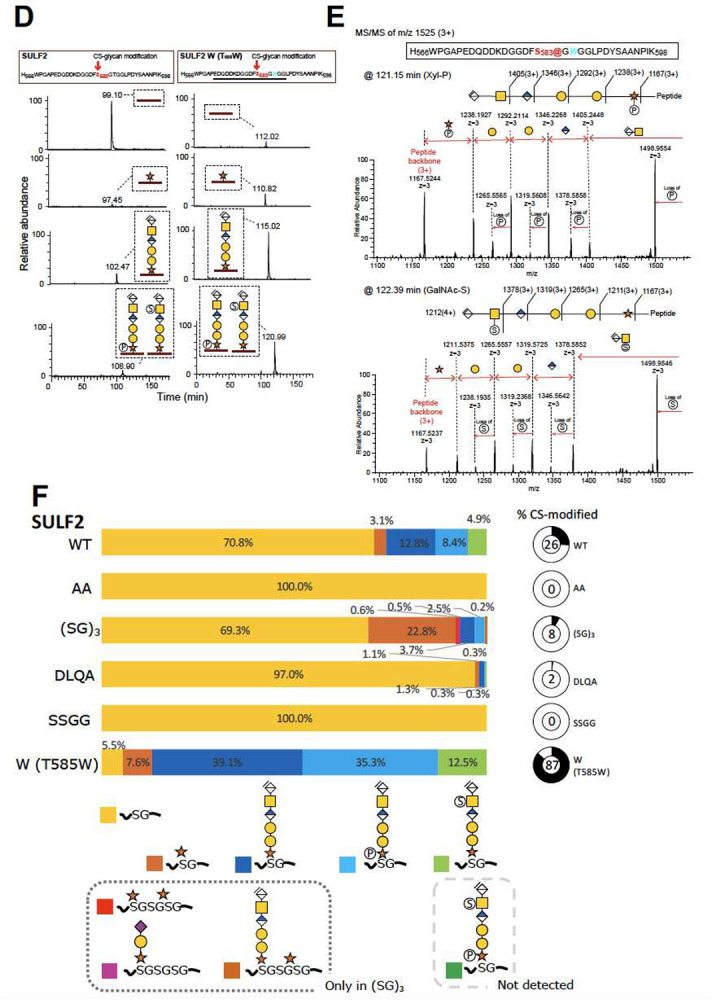
The amino acid sequences required for chondroitin sulfate (CS) biosynthesis and its strong induction by a tryptophan substitution in SULF2. (**A**) Schematic representation of the SULF2 protein. Red and light blue boxes indicate the sulfatase domain and hydrophilic domain, respectively. Arrows with dashed lines indicate furin cleavage sites. CS-glycosaminoglycan (GAG) modification occurs at Ser^583^ in human SULF2. (**B**) A three-dimensional model of the structure of human SULF2 predicted by AlphaFold v2. Blue indicates a per-residue confidence score (pLDDT) greater than 90. Light blue indicates a score greater than 70. Yellow indicates a score greater than 50. Orange indicates a score less than 50^51^. The positions of the signal peptide (SP) and furin-type cleavages, as well as the CS-GAG modification, are shown. The coiled-coil structural units that are potential multimerization elements in the hydrophilic domain are shown by an open arrowhead. The positions of the first Met^1^ and the last Gly^870^ are indicated by blue circles with dashed lines. (**C**) Amino acid sequences of the original and variant forms of the SULF2 protein. The sequences shown in pink and purple are the original sequence of SULF2. Light green and green show the original sequence of SULF1. The concentrated CM from the transfected HEK293 cells was subjected to Western blotting to detect the His tag. Full-length SULF2, with or without CS, was detected ≥250 kDa and 130 kDa, respectively. The CS-modified C-subunits, which are produced after furin cleavage, were detected at around 95–180 kDa, while the C-subunits without CS were found at 45–55 kDa in several forms. In the (SG)_3_ variant, the CS-modified C-subunit is also observed at ≥250 kDa. The schematic representation of the predicted major components of the SULF2 subunits is shown on the right. The intensity of the anti-His signal was quantified (n = 7). Data are means ± SE. *P < 0.05, **P < 0.01, ****P < 0.0001. (**D**) Extracted ion chromatograms of CS-glycopeptides that are glycosylated at Ser^583^. Representative data in SULF2 and the SULF2 W(T_585_W) variant are shown. SULF2 samples were digested with chondroitinase ABC followed by on-bead trypsin digestion and C18 reversed-phase nano LC–MS/MS analysis. The extracted ion chromatograms of the indicated peptides and glycopeptides are shown. The peptide backbone sequences are indicated. **(E)** The tandem mass (MS/MS) analysis of CS-core oligosaccharides with mono-phosphorylation or mono-sulfation. Representative data in the SULF2 W(T_585_W) variant are shown. MS/MS spectra of two representative CS-glycopeptides identified in the SULF2 W(T_585_W) variant are shown: a CS-glycopeptide that is phosphorylated on xylose (upper spectrum) and a CS-glycopeptide that is sulfated on GalNAc (lower spectrum). The base peak at m/z 362 corresponds to the UHexA–HexNAc disaccharide at the non-reducing end of the CS core while m/z 442 (UHexA–HexNAc–Sulfate) indicates sulfation on the disaccharide. The fragment at m/z 1356 indicates xylose phosphorylation. The peptide backbone sequence of each glycopeptide is highlighted in a box. The glycosylation site is indicated by “@” and the mutated residue is shown in italics. Monosaccharides are depicted using the Symbol Nomenclature for Glycans (SNFG, ver 1.5). A discovered CS sequon is highlighted with an underline. (**F**) Relative peak intensity comparison (%) of the CS-modified glycopeptides and the CS-unmodified peptides determined by nano LC-MS/MS. The CS-glycopeptides shown in the dashed circle were only found in the (SG)_3_ variant. The CS-glycopeptides having both mono-phosphate and mono-sulfate were not detected in any of the SULF2 proteins. The percentage of the CS modification found in SULF2 and its variants is indicted on the right.

The pre-proprotein SULF2 consists of a signal peptide, two sulfatase domains, and a hydrophilic domain. After the signal peptide is removed, the proprotein undergoes processing by a furin-type protease at the Arg^538^ and Arg^565^ sites within the hydrophilic domain. This process yields 75-kDa N- and 50-kDa C-subunits that are linked by disulfide bonds^1, 13^. SULF2 but not SULF1, a related endoglucosamine-6-sulfatase family member, is modified with CS at Ser^583^ in the hydrophilic domain (Fig. 1, A and B). To elucidate the mechanism by which CS modification occurs in SULF2 but not in SULF1, we transiently expressed the C-terminal His-tagged human SULF2 and various mutated variants in human HEK293 cells. Their secreted forms in the conditioned medium (CM) were analyzed by Western blotting with an anti-His antibody (Fig. 1C). As previously reported^1, 13^, a portion of the transiently expressed recombinant SULF2 exhibits a polydisperse (smeared) signal with molecular weight (MW) of 95–180 kDa, which corresponds to the C-subunit with CS modification of SULF2^2^. This polydisperse high MW signal was not observed when Ser^583^ was replaced with Ala together with an Ala substitution for the neighboring Gly^584^ (SULF2 AA), nor when nine consecutive amino acid residues containing Ser^583^ (^579^Gly-Gly-Asp-Phe-Ser-Gly-Thr-Gly-Gly^587^; ^579^GGDFSGTGG^587^) were replaced with the corresponding nine amino acids of human SULF1 (^590^Ser-Ser-Gly-Gly-Asn-Arg-Gly-Arg-Met^598^; ^590^SSGGNRGRM^598^) (SULF2 SSGG). The signal of the BE-123 antibody binding, which recognizes the CS stub epitope generated by chondroitinase ABC (ChABC) digestion, appeared in the SULF2 sample after treatment with ChABC, whereas no signal was seen in the SULF2 AA mutant sample, confirming that the modification is the addition of CS and not another GAG such as HS (**fig. S1**). SULF2 is modified with N-glycans both in the N- and C-subunits^1, 14, 15^. De-N-glycosylation with PNGaseF yielded the expected lower MW forms of both subunits in wildtype and the SULF2AA variant, indicating that the CS sequon did not influence N-glycosylation (**fig. S2**).

Previous studies have shown that adjacent Ser-Gly (SG) repeats can enhance the attachment of GAG chains^8^. We wished to determine whether inserting three SG repeats adjacent to Ser^583^ in SULF2 would produce a stronger high MW polydisperse signal of the same molecular size as a result of having more available triggers for CS modifications (SULF2 (SG)_3_). High MW polydisperse SULF2 was observed for expression of the SULF2 (SG)_3_ construct, but contrary to what was expected, its size was not 95–180 kDa but rather a larger size of 250 kDa or more. To confirm that the high MW signals observed in the SULF2 and SULF2 (SG)_3_ mutants were due to the attachment of CS chains, we pretreated these protein samples with ChABC, followed by blotting with the 2B4 anti-SULF2 C-subunit antibody^16^. We found that the polydisperse signals with MW of 95–180 kDa and ≥ 250 kDa disappeared after treatment with ChABC in these variants (**fig. S3**). Pretreatment with a mixture of heparinases I, II, and III did not affect the intensity or size of the signals in these variants. Thus, for SULF2, adding SG repeats did not result in the conversion of CS synthesis to HS synthesis^17^. Adding single or double SG produced similar or lower MW signal levels as compared to the (SG)_3_ mutant variant (**fig. S3**).

SULF2 has a unique cluster of seven amino acid residues including five acidic and one basic residue in the immediate vicinity of Ser^583^ (^572^Glu-Asp-Gln-Asp-Asp-Lys-Asp^578^; ^572^EDQDDKD^578^). To elucidate the potential regulatory role of the charged cluster ^572^EDQDDKD^578^, we replaced it with the amino acid sequence of the corresponding site of the SULF1 protein (^583^Gly-Pro-Arg-Asp-Leu-Gln-Ala^589^; ^583^GPRDLQA^589^) (SULF2 DLQA). The DLQA variant produced comparable results to those of the AA variant of SULF2, suggesting that the CS addition is negligible and that the ^572^EDQDDKD^578^ cluster is a prerequisite for CS modification in SULF2 (Fig. 1C **and fig. S4**). It is known that aromatic amino acids located near the Ser residues undergoing GAG modification enhance GAG assembly^7^. We asked whether introducing a tryptophan (Trp) next to Ser^583^ would impact the degree of CS modification. We replaced Thr^585^ with Trp (SULF2 W(T_585_W)). The SULF2 W(T_585_W) variant showed a significantly higher level of the high MW polydisperse signals at 95–180 kDa MW than the non-mutated form of SULF2 (Fig. 1C **and fig. S4**). In rat betaglycan, the eight amino acid sequence (^538^Pro-Asp-Gly-Tyr-Glu-Asp-Leu-Glu^545^; ^538^PDGYEDLE^545^), which is located near the Ser^535^ undergoing GAG modification, is necessary for GAG assembly^7^. We tested whether introducing this amino acid sequence would show additional enhancement of CS synthesis in SULF2 (SULF2 W-PDGY (T_585_W-PDGY)). The results for the SULF2 W-PDGY (T_585_W-PDGY) variant were comparable to those for SULF2 W (T_585_W) (**fig. S4**), indicating that insertion of the PDGYEDLE sequence has no significant effect on CS synthesis in SULF2.

To determine how substitution and introduction of the aforementioned amino acid sequence influences CS modification and to quantify CS addition, purified SULF2 and its variants were analyzed by nano liquid chromatography (LC)-tandem mass spectrometry (MS/MS)^18, 19^ following ChABC digestion. ChABC removes the polymerized CS chain while preserving the residual linkage region glycan covalently attached to the Ser residue^20^. This treatment converts the heterogeneous CS polymer into a defined residual structure containing a Δ^4^-unsaturated uronic acid at the non-reducing end, which serves as a diagnostic signature for CS-derived glycopeptides in MS/MS analysis (**fig. S5**). This treatment markedly improved reversed-phase retention and ionization efficiency of CS glycopeptides and enabled detailed structural interrogation of the CS linkage region. Most CS glycopeptide species were chromatographically resolved. A critical exception was species containing either a single phosphate on xylose or a single sulfate on GalNAc of the linkage region. These two species have nearly identical precursor masses due to the close masses of phosphate (ΔM = 79.9663 Da) and sulfate (ΔM = 79.9568 Da). However, these species were unambiguously distinguished by their MS/MS fragmentation behavior, and assignments were manually validated based on diagnostic fragment ions characteristic of each modification (Fig. 1, D and E). Xylosyl-phosphorylated glycopeptides generated the fragment ions corresponding to the peptide backbone carrying a xylose and a phosphate moiety, confirming phosphorylation at the xylose residue. In contrast, GalNAc-sulfated species produced characteristic neutral losses and fragment ions corresponding to the sulfated UHexA–HexNAc disaccharide at m/z 442. These species were partially co-eluted, with the xylose-phosphorylated form eluting slightly earlier than the GalNAc-sulfated form (Fig. 1, D and E). Using this approach, the non-mutated form of SULF2 expressed transiently in HEK293 cells exhibited 26% CS modification at Ser^583^, approximately 3% mono-xylosylation at the same residue, and 71% unmodified peptide (Fig. 1F **and table S1**). SULF2 AA and SULF2 SSGG variants showed no detectable CS modification or mono-xylosylation at Ser^583^. SULF2 DLQA showed only ~2% CS modification, supporting the requirement of the acidic cluster ^572^EDQDDKD^578^ for CS initiation. The SULF2 (SG)_3_ variant, which by SDS–PAGE appeared to exhibit enhanced glycosylation with high MW bands above 250 kDa, showed substantially lower CS occupancy than anticipated. Only 8% of peptides were CS-modified at Ser^583^, while approximately 70% remained unmodified. Notably, mono-xylosylated peptide increased to 22% (Fig. 1F **and table S1**). It has been shown that the insertion of SG repeats does not promote the formation of additional CS initiation sites. Rather, it impairs efficient progression beyond the initial xylose transfer step. The accumulation of mono-xylosylated species strongly suggests that galactosylation is defective during the formation of the tetrasaccharide linkage region in this variant. Therefore, the pronounced MW shift observed by Western blotting is best explained by the elongation of CS chains at a limited number of sites where CS modification is successfully initiated, rather than by an increased number of CS-modified sites per protein. This interpretation is supported by the detection of low-level mono-xylosylation on the introduced SG repeat serines (0.6% ± 0.3%) and the observation of a rare xylose–galactose–sialic acid structure (0.5%) (Fig. 1F, **fig. S6, and table S1**), which is consistent with the presence of premature or stalled linkage structures in the (SG)_3_ variant. The reason for sialic acid-mediated termination of the elongation remains unknown^21^. In contrast, the SULF2 W(T_585_W) variant showed nearly 90% CS modification at Ser^583^, with distinct subpopulations of linkage region structures containing either xylose phosphorylation or GalNAc sulfation. No glycopeptides containing both modifications were detected (Fig. 1, D to F, **and table S1**). The high proportion of xylose-phosphorylated species suggests that removal of the phosphate by phosphoxylose phosphatase 1 (PXYLP1) is not a prerequisite for continued CS polymerization at this site. Further analysis is required to determine its regulatory role in CS chain length^22, 23^. These results demonstrate that Trp substitution near the glycosylated Ser residue strongly enhances efficient progression of CS synthesis following xylose transfer.

### Validation of the CS Sequon: Amino acid sequence sufficient for triggering a novel CS modification in SULF1

**Fig. 2.**
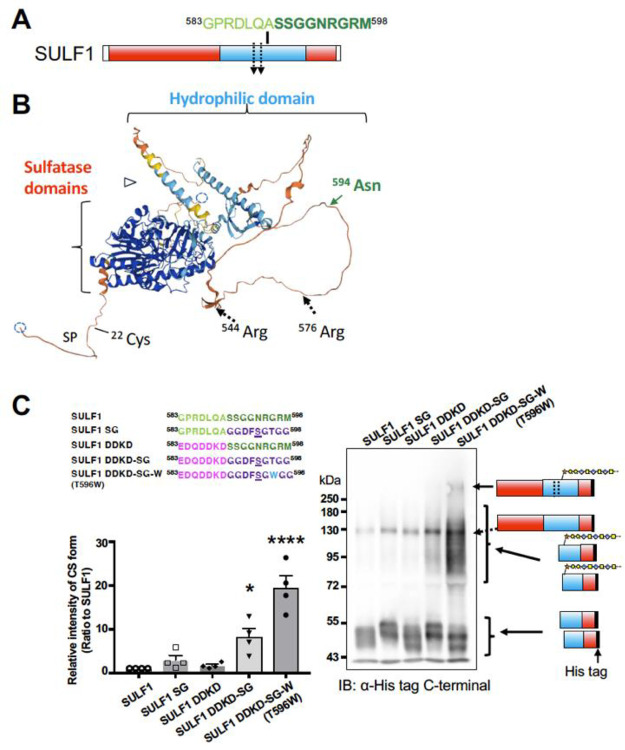

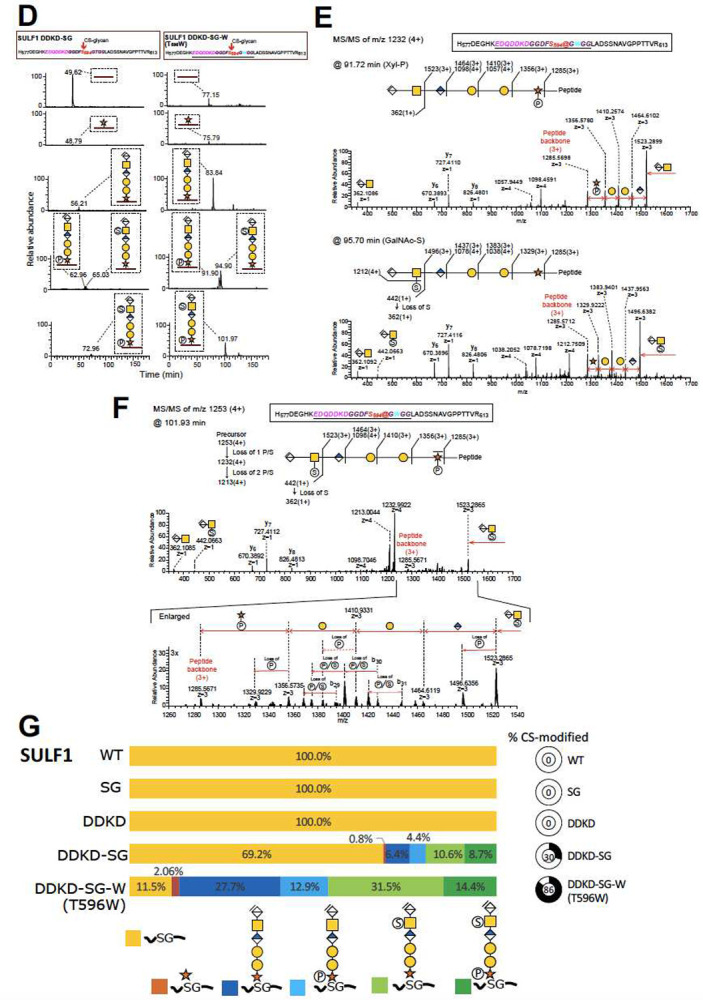
Development of a novel CS-modified SULF1 and identification of a sequon, EDQDDKDGGDFSGWGG, adequate for efficient CS biosynthesis. (A) Schematic representation of the SULF1 protein. The red and light blue boxes show the sulfatase and hydrophilic domains, respectively. Arrows with dashed lines indicate furin cleavage sites. The amino acid sequence of the SULF1 protein that corresponds to the CS modification site of the SULF2 protein is shown. (**B**) A three-dimensional model of the structure of human SULF1 predicted by AlphaFold v2. Blue indicates a per-residue confidence score (pLDDT) greater than 90. Light blue indicates a score greater than 70. Yellow indicates a score greater than 50. Orange indicates a score less than 50^51^. The positions of the signal peptide (SP) and furin-type cleavages are shown. The position of Asn^594^ corresponds to Ser^583^ in SULF2. The coiled-coil structural units that are potential multimerization elements in the hydrophilic domain are shown by an open arrowhead. The positions of the first Met^1^ and the last Gly^871^ are indicated by blue circles with dashed lines. (**C**) Amino acid sequences of the original and variant forms of the SULF1 protein. The sequences shown in green and light green are the original sequence of SULF1. Pink and purple show the original sequence of SULF2. The SULF1 DDKD-SG-W (T_596_W) variant harbors the CS sequon, EDQDDKDGGDFSGWGG. The concentrated CM from the transfected HEK293 cells was subjected to Western blotting to detect the His tag. Full-length SULF1, with or without newly synthesized CS, was detected ≥250 kDa and 130 kDa, respectively. The CS-modified C-subunits, which are produced after furin cleavage, were detected at around 95–180 kDa, while the C-subunits without CS were found at 45–55 kDa in several forms. The schematic representation of the predicted major components of the SULF1 subunits is shown on the right. The intensity of the anti-His signal was quantified (n = 4). Data are means ± SE. *P < 0.05, ****P < 0.0001. (**D**) Extracted ion chromatograms of CS-glycopeptides that are glycosylated at Ser^594^. Representative data in the SULF1 DDKD-SG and SULF1 DDKD-SG-W (T_596_W) variants are shown. SULF1 samples were digested with chondroitinase ABC followed by on-bead trypsin digestion and C18 reversed-phase nano LC–MS/MS analysis. The extracted ion chromatograms of the indicated peptides and glycopeptides are shown. The peptide backbone sequences are indicated. **(E, F)** The tandem mass (MS/MS) analysis of CS-core oligosaccharides with mono-phosphorylation, mono-sulfation, or both. Representative data in the SULF1 DDKD-SG-W (T_596_W) variant are shown. MS/MS spectra of three representative CS-glycopeptides identified in the SULF1 DDKD-SG-W (T_596_W) variant are shown: a CS-glycopeptide that is phosphorylated on xylose (upper spectrum in E), a CS-glycopeptide that is sulfated on GalNAc (lower spectrum in E), and a CS-glycopeptide that carries both mono-phosphorylated xylose and mono-sulfated GalNAc (spectrum in F). The base peak at m/z 362 corresponds to the UHexA–HexNAc disaccharide at the non-reducing end of the CS core while m/z 442 (UHexA–HexNAc–Sulfate) indicates sulfation on the disaccharide. The fragment at m/z 1356 indicates xylose phosphorylation. The peptide backbone sequence of each glycopeptide is highlighted in a box. The glycosylation site is indicated by “@” and the mutated residues are shown in italics. Monosaccharides are depicted using the Symbol Nomenclature for Glycans (SNFG, ver1.5). A discovered CS sequon is highlighted with an underline. (**G**) Relative peak intensity comparison (%) of the CS-modified glycopeptides and the CS-unmodified peptides determined by nano LC- MS/MS. The CS-glycopeptides having both mono-phosphate and mono-sulfate were detected in the new CS-modified forms of SULF1 proteins. The percentage of the CS modification found in SULF1 and its variants is indicated on the right.

As in the case of SULF2, SULF1 is processed by furin and secreted extracellularly^1, 13, 24^. In contrast to SULF2, SULF1 is not CS-modified (Fig. 2, A and B). C-Terminal His-tagged human SULF1 and its variants transiently expressed in HEK293 cells were purified from the CM and then analyzed by Western blotting. SULF1 is modified with N-glycans both in the N- and C-subunits^1^ (**fig. S7**). Replacing nine consecutive amino acid residues (^590^SSGGNRGRM^598^) of SULF1 with the corresponding portion (^579^GGDFSGTGG^587^) of SULF2 that contains the CS-attachment site Ser (SULF1 SG), or replacing the ^583^GPRDLQA^589^ residues of SULF1 with the acidic amino acid cluster (^572^EDQDDKD^578^) of SULF2 (SULF1 DDKD), did not produce high MW signals (90–180 kDa) probing with anti-His antibody (Fig. 2C, **and fig. S8**). When we replaced both of these amino acid sequences in SULF1 (SULF1 DDKD-SG), a modest amount of the high MW signal was observed which was eliminated by ChABC pretreatment (**fig. S8**). BE-123 CS stub epitope was observed in SULF1 DDKD-SG after ChABC digestion, indicating that new CS addition had been initiated and elongated (**fig. S8**). The variant (SULF1 DDKD-SG-W (T_596_W)) obtained by adding a Trp substitution to the DDKD-SG variant exhibited a distinct, very strong high MW polydisperse signal at the 90–180 kDa size (Fig. 2C, **and fig. S8**). Thus, introducing Trp near the Ser residue undergoing CS modification dramatically enhances CS synthesis in the SULF1 DDKD-SG-W (T_596_W) variant, as was observed in the SULF2 W(T_585_W) variant. Insertion of the eight amino acid sequence PDGYEDLE (SULF1 DDKD-SG-W-PDGY (T_596_W-PDGY)) resulted in the signals comparable to SULF1 DDKD-SG-W (T_596_W) mutant (**fig. S8**). These results indicated that the insertion of 16 amino acid sequence EDQDDKDGGDFSGWGG is sufficient to trigger and initiate new post-translational CS modification in SULF1. Hence, we have discovered a new functional CS sequon, EDQDDKDGGDFSGWGG, that directs CS addition to an extracellular protein. Whether the discovered CS sequon functions only in the context of residing within an intrinsically disordered region, such as the hydrophilic domain of the SULFs, remains to be determined^25^.

The same LC–MS/MS workflow was applied to SULF1 and its variants. No detectable CS modification, truncated linkage structures, or mono-xylosylation was seen in SULF1, SULF1 SG, or SULF1 DDKD, consistent with the observation that modification of either the acidic cluster or the Ser-Gly region alone is insufficient to initiate CS biosynthesis (Fig. 2, D to G, **and table S2**). The results for the SULF1 SG variant were consistent with the finding in SULF2 that the acidic amino acid cluster EDQDDKD is crucial for CS synthesis. The combined substitution variant, SULF1 DDKD-SG, resulted in clear CS modification at the introduced Ser^594^ with an occupancy of approximately 30%, comparable to the non-mutated SULF2. A low level of mono-xylosylated peptide (~0.8%) was also detected, confirming the partial initiation of the linkage region (Fig. 2, D to G, **and table S2**). Consistent with the biochemical observations, introduction of Trp (T_596_W) in the SULF1 DDKD-SG variant increased CS modification at Ser^594^ to ~86%, with ~2% mono-xylosylated species still detectable. Notably, unlike SULF2 and its variants, the CS-engineered SULF1 proteins exhibited linkage-region glycopeptides containing both xylose phosphorylation and GalNAc sulfation (Fig. 2, D to G, **and table S2**). This distinct structural pattern indicates that, although the 16-amino-acid CS-sequon EDQDDKDGGDFSGWGG is sufficient to initiate CS modification at the introduced Ser, additional sequence elements outside the sequon in SULF1 influence subsequent processing of the linkage region.

### Functional CS Sequon controls xylosyltransferase activity

**Fig. 3.**
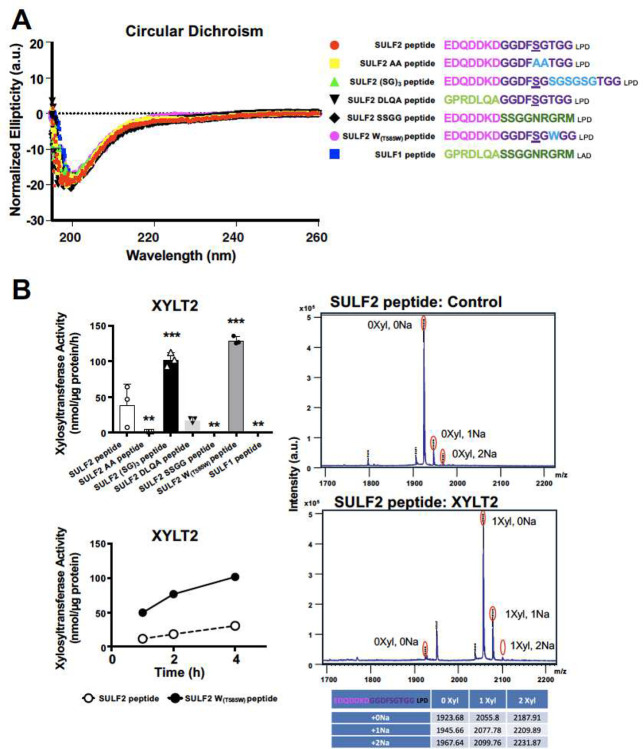
The CS sequon EDQDDKDGGDFSGWGG controls xylosyltransferase 2 activity. (**A**) Secondary structure of the peptides revealed by circular dichroism (CD) analysis. Normalized far-UV CD spectra of the synthesized SULF variant peptides are shown. These peptides form similar random coil structures. The SULF2 W (T_585_W) peptide harbors the CS sequon, EDQDDKDGGDFSGWGG. (**B**) In vitro assay of xylosyltransferase 2 (XYLT2). XYLT2 activity is selectively higher with the SULF2 (SG)_3_ and W (T_585_W) peptides compared to that with the SULF2 peptide (n = 3). Columns with mean values less than 0.01 in the SULF2 SSGG peptide and the SULF1peptide. Data are means ± SE. **P < 0.01, ***P < 0.001. The preferential activity of XYLT2 increases over time with the SULF2 W (T_585_W) peptide. Monoxylosylation of the SULF2 peptide was verified using MALDI-TOF MS analysis.

To investigate whether the secondary structure of the consecutive 16-amino acid sequence of the CS sequon controls the initiation and elongation of CS biosynthesis, interactions with the driving force of aromatic rings were analyzed. These interactions include those between amino acid side chains, such as cation/anion-π and π-π interactions^26, 27^. We synthesized a series of peptides derived from these sequences and then analyzed their secondary structure and folding properties using circular dichroism (CD) spectroscopy^28^. CD measurements revealed a comparable pattern of differential absorption of left- and right-circularly polarized light, suggesting that the random coil structures of all peptides derived from SULF2, its variants, and SULF1 are indistinguishable (Fig. 3A). We also focused on two residues: Lys^577^, a unique positively charged residue in the ^572^EDQDDKD^578^ cluster; and Phe^582^, an aromatic residue in the vicinity of Ser^583^. Western blotting patterns of the mutated SULF2 variants (SULF2 K_577_A and SULF2 F_582_A) and the CD measurements of the synthetic peptides containing each amino acid substitution were comparable to those of non-mutated SULF2 (**fig. S9, A and B**), indicating that differences in the secondary structure surrounding the CS-modified Ser residue were not the controlling factor for CS modification of the SULF proteins. Next, we verified that the functional CS sequon controls the transfer of xylose to the Ser residue, which is the first step of CS modification^10^. This step is catalyzed by either xylosyltransferase 1 (XYLT1) or xylosyltransferase 2 (XYLT2)^29^. We developed a new XYLT activity assay utilizing recombinant human XYLT1 or XYLT2, the xylose donor uridine diphosphate (UDP)-xylose, and a luminescence probe that detects UDP. Since HEK293 cells predominantly express XYLT2, but not XYLT1 (**fig. S10**), we tested the synthesized peptides using XYLT2 in the assay. Consistently, XYLT2 exhibited xylose transfer activity toward the SULF2 peptide but showed no activity toward the SULF1 peptide (Fig. 3B). The peptides derived from the SULF2 AA or SULF2 SSGG variant were not substrates for the enzyme. The SULF2 DLQA peptide showed a subtle activity. The SULF2 (SG)_3_ peptide showed a 1.8-fold increase as a substrate for XYLT2. In good agreement with the results of the biochemical analysis and LC-NSI-MS/MS described above, the SULF2 W (T_585_W) peptide showed a 3-fold increase as a substrate. This increase was time-dependent (Fig. 3B). We also measured the activity of XYLT1 toward these peptides. The results showed a slight preference for the SULF2 (SG)_3_ peptide and the SULF2 W (T_585_W) peptide, but not to the same extent as for XYLT2. The increased activity toward the SULF2 W (T_585_W) peptide was not accelerated over time (**fig. S11**). The activity of either XYLT2 or XYLT1 toward the peptides derived from the SULF2 K_577_A and SULF2 F_582_A variants was comparable to the activity against the peptide of SULF2 (**fig. S12**). These results strongly suggest that the dramatic increase in CS synthesis upon introduction of Trp substitution in HEK293 cells likely occurred through an XYLT2-dependent mechanism.

### CS addition increases the extracellular abundance of the modified SULF proteins and their endoglucosamine-6-sulfatase activity

**Fig. 4.**
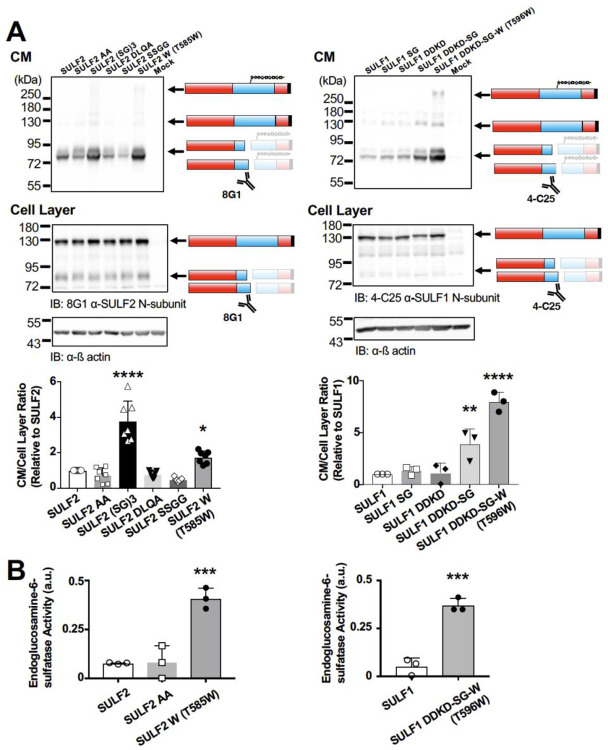

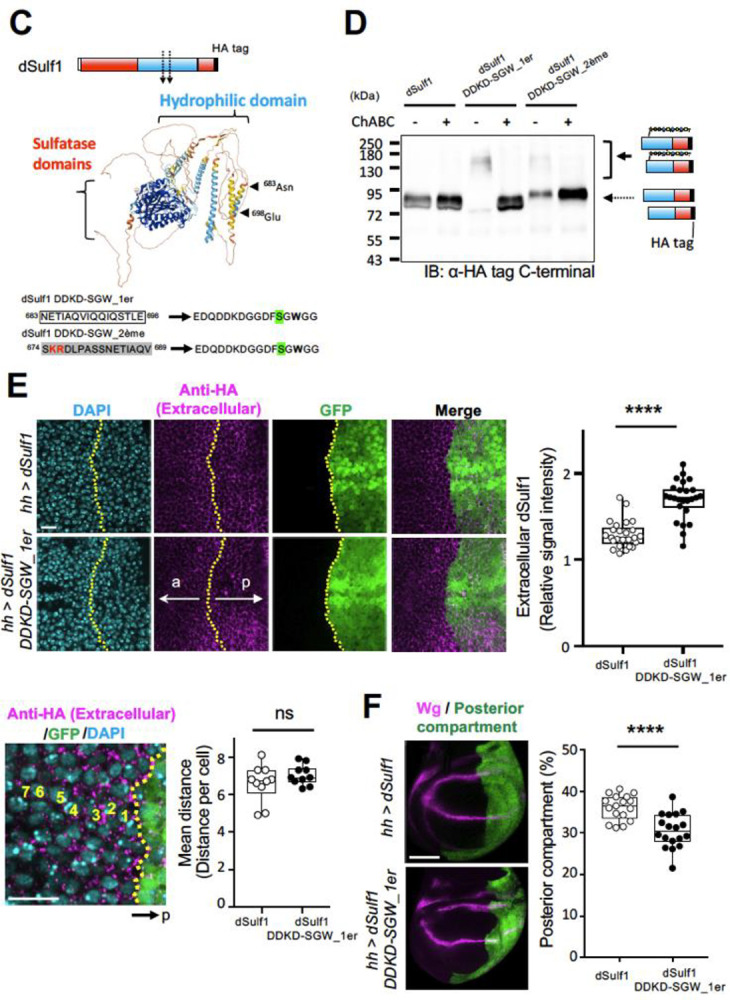
Increased CS modification levels correlate with higher amounts of extracellular SULF proteins and greater endoglucosamine-6-sulfatase activity, and the CS sequon is also functional in Drosophila. (**A**) Immunoblotting analysis of expressed SULF proteins in conditioned medium (CM) and cell lysate using the following antibodies: 8G1 for the SULF2 variants and 4-C25 for the SULF1 variants. The variants, the SULF2 W (T_583_W) and the SULF1 DDKD-SG-W (T_596_W), harbor the CS sequon, EDQDDKDGGDFSGWGG. The signal intensities of each variant’s bands were quantified, and the CM/cell lysate ratio of the intensities was calculated (n = 7 for the SULF2 group and n = 3 for the SULF1 group). The schematic representation of the predicted major components of the subunits is shown on the right. Data are means ± SE. *P < 0.05, **P < 0.01, ***P < 0.001. (**B**) The endoglucosamine-6-sulfatase activities of the SULF2 and SULF1 variants were measured using an ELISA (n = 3). (**C**) Integration of the CS sequon EDQDDKDGGDFSGWGG into Drosophila Sulf1 (dSulf1). Red and light blue boxes indicate the sulfatase domain and hydrophilic domain, respectively. Arrows with dashed lines indicate furin cleavage sites. A three-dimensional model of the structure of dSulf1 predicted by AlphaFold v2^51^. The amino acid positions from the start to the end of the region substituted in dSulf1 DDKD-SGW-1er are indicated by arrowheads. The substituted positions with the CS sequon in the dSulf1 DDKD-SGW-1er and dSulf1 DDKD-SGW-2ème variants are shown. The boxed and shaded positions in the entire dSulf1 are shown in **figure S17**. A portion of the consensus furin cleavage sites with basic amino acids in red are indicated. The Ser corresponding to Ser^583^ of SULF2 in the CS sequon is highlighted in green. (**D**) Immunoblotting to detect CS modification in the CS sequon-integrated dSulf1. A polydisperse signal ranging from 120 to 200 kDa appeared in the dSulf1 DDKD-SGW-1er and dSulf1 DDKD-SGW-2ème variants. Pretreatment with ChABC cleared these bands and restored the detection of the 90-kDa bands. The degree of CS biosynthesis varies according to the positions of the CS sequon. (**E**) The CS sequon functions in the Drosophila wing disc in vivo. Drosophila strains with either the UAS-dSulf1 transgene (dSulf1) or the UAS-CS sequon-integrated dSulf1 transgene (dSulf1 DDKD-SGW-1er) were generated. Expression of the transgenes was induced in the posterior compartment using a hedgehog (hh)-Gal4 driver. DAPI was used to show cell nuclei. Transgene-derived dSulf1 proteins in the anterior (a) and posterior (p) compartments are revealed by extracellular immunostaining in wing discs (magenta). The posterior compartment is marked by GFP expression (green). Extracellular dSulfs within the posterior compartment of the wing pouch were quantified (n = 25 for each). The spread distance that dSulf1 extends from the boundary of GFP-positive region in the wing pouch was measured and quantified (n = 10 for each). Scale bar: 10 μm. (**F**) The activity of dSulfs in the wing disc in vivo. The activity of dSulfs was quantified by measuring the proportion of the posterior compartment expressing GFP (green) within the entire wing pouch (n = 17 for dSulf1, n = 18 for dSulf1 DDKD-SGW-1er). Extracellular Wg was immunostained (magenta). The posterior compartment’s size is inversely proportional to the activity of dSulf1^35, 36^. The data are presented in the form of a box-and-whisker diagram. ns: not significant. ****P < 0.0001. Scale bar: 100 μm.

Next, we utilized the discovered CS sequon to analyze the function of CS addition in SULFs by examining whether CS modification influences SULF protein secretion^1^. The amount of SULF proteins found in CM or in the cultured cell layer of cells expressing SULF mutants was measured by immunoblot using the 8G1 and 4-C25 antibodies^30, 31^, which recognize the N-subunit epitopes of SULF2 and SULF1, respectively. The ratios of CM to cell layer SULF abundance was calculated and found to be 2- to 3-fold higher in the CS-modified variants, SULF2 (SG)_3_ and SULF2 W(T_585_W), than in non-mutated SULF2 (Fig. 4A). Conversely, in SULF2 AA, DLQA, and SSGG, where CS modification did not occur or was low, the CM/Cell layer ratio decreased. In the CS-modified SULF1 DDKD-SG and SULF1 DDKD-SG-W (T_596_W) variants, the CM/cell layer ratio increased 4- and 8-fold, respectively, compared to the unmodified SULF1 (Fig. 4A). These results indicate that CS modification promotes the extracellular secretion of the modified proteins, thereby increasing the abundance of the CS-modified SULF protein in the CM. We next tested whether CS modification influences the HS endoglucosamine-6-sulfatase activity of the secreted SULFs by an ELISA method^32, 33^. We found that the activities of the CS-modified variants of SULF2 W (T_585_W) and SULF1 DDKD-SG-W (T_596_W) were significantly higher than those of their CS-unmodified counterparts (Fig. 4B). These results indicate that CS modification enhances SULF endosulfatase activity.

The SULF pro-proteins are cleaved by a furin-type convertase into two subunit chains forming disulfide-linked heterodimers^1, 13^. The double disulfide bonds between the two C-X-C motifs within the hydrophilic domain are thought to be the primary bonds connecting the heterodimers in the SULF family^13^. Indeed, mutations in one of these motifs disrupted the formation of the N- and C-subunit linkage (**fig. S13**). We analyzed whether the CS modification influences the formation of higher-order SULF2 proteoforms by creating new mutated variants with deletion of the two furin-cut sites, ^533^RSRSIR^538^ and ^560^RNLTKR^565^ (SULF2 D1aD2). Under reducing conditions (with β-mercaptoethanol, β-ME), the protein band with a MW greater than 250 kDa was not observed in a furin-uncleavable CS-unmodified variant (SULF2 AA_D1aD2) (**fig. S14**). Interestingly, this protein band signal increased in a furin-uncleavable, CS-modified variant (SULF2 W(T_585_W)_D1aD2). Under non-reducing conditions (without β-ME), both the AA and AA_D1aD2 variants of SULF2 showed bands >250 kDa with both the 8G1 and anti-His antibodies (**fig. S14**). The results for the SULF2 D1aD2 furin-uncleavable variant were confirmed in another substitution mutation, R_538_A/KR_564,565_AA (**fig. S15**). These results indicate that CS modification increases the formation of higher-order SULF2. The enhancement of multimer formation due to CS addition appears to have a stronger effect on the furin-uncleaved forms. Higher-order SULF multimers may form via the coiled-coil motif in the hydrophilic domain^1, 13, 25^. The precise impact of the CS chain on SULF molecular interactions mediated by the coiled-coil domain remains a subject for future research. The SULF endoglucosamine-6-sulfatase activity may be enhanced by higher-order formation, which is intrinsically regulated by CS modification.

Similar to the results observed in HEK293 cells, higher-order bands ≥250 kDa and polydisperse material of lower MW (between 95–180 kDa) were detected in transfected CHO cells using an anti-His antibody for SULF2 (SG)_3_, SULF2 W(T_585_W), SULF1 DDKD-SG, and SULF1 DDKD-SG-W (T_596_W) variants (**fig. S16A**). In the SULF2 W (T_585_W) and SULF1 DDKD-SG-W (T_596_W) variants, a 130 kDa band remained detectable in CHO cells. This result indicates that the effects of Trp substitution on CS initiation and elongation in CHO cells is slightly lower than observed in HEK293 cells. Furin-mediated maturation is expected to occur at a lower level in CHO cells than in HEK293 cells. Consistent with this expectation, the 130 kDa SULF bands and their variants appear more pronounced in CHO cells than in HEK293 cells. While further clarification of the regulation of furin susceptibility of the SULFs is required, both human SULF1 and SULF2 may exhibit reduced membrane microdomain localization upon expression in the rodent-derived CHO cells^13^. Consistent with the findings in HEK293 cells, SULF2 AA protein secretion decreased while (SG)_3_ protein secretion increased into the CM of expressing CHO cells (**fig. S16B**), indicating common functions of the CS modification across mammalian cells.

### The CS sequon is also functional in Drosophila

Next, we verified whether the CS sequon functions in a non-mammalian, invertebrate SULF. As first demonstrated in quail SULF1^34^, drosophila SULF1 (dSulf1) regulates Wingless signaling and is the sole homologue of the human SULFs in flies^35, 36^. dSulf1 possesses a hydrophilic domain approximately 200 amino acid residues longer than that of human SULFs (Fig. 4C **and fig. S17**). We introduced the discovered CS sequon, EDQDDKDGGDFSGWGG, into the hydrophilic domain of HA-tagged dSulf1. The consecutive 16 amino acid residues (^683^NETIAQVIQQIQSTLE^698^), which reside in the intrinsically disordered region of the hydrophilic domain and are proximal to the second furin cleavage site of dSulf1, were replaced (dSulf1 DDKD-SGW_1er). We also expressed another variant in which the amino acid residues including the second furin cleavage site (^674^SKRDLPASSNETIAQV^689^) were replaced (dSulf1 DDKD-SGW_2ème) (Fig. 4C **and fig. S17**). Interestingly, the DDKD-SGW_1er variant showed a polydisperse signal at 120–200 kDa that was not detected in dSulf1 (Fig. 4D **and fig. S18A**). Concomitantly, detection of the 90 kDa bands was almost absent for the DDKD-SGW_1er variant. The polydisperse signal disappeared upon ChABC treatment, and detection of the 90 kDa bands was restored, indicating that CS modification occurred on the DDKD-SGW_1er variant (Fig. 4D **and fig. S18A**). Similar results were observed in the DDKD-SGW_2ème variant, but the degree of CS modification was found to be lower compared to the DDKD-SGW_1er variant (Fig. 4D). These results demonstrate that the discovered CS sequon is functionally active in the drosophila protein. The efficiency of successful CS modification induction was impacted by differences in the insertion site and the CS sequon function exhibited by this dSulf1 DDKD-SGW_1er variant was observed not only in HEK293 cells but also in drosophila Dmel2 cells (**fig. S18B**).

We asked whether the CS sequon is functional in vivo. We generated a drosophila strain harboring the dSulf1 DDKD-SGW_1er transgene under the UAS promoter. Expression of the CS-sequon-integrated dSulf1 was driven by the hedgehog (hh)-GAL4 driver. The UAS transgenes were expressed in the posterior compartment of the wing disc by crossing the hh-Gal4 strain. The posterior compartment of the wing disc was visualized using the GFP transgene, which is regulated by the UAS promoter^36^. To determine if CS synthesis controls the presence of dSulf1 proteins in the extracellular space in vivo, we used a method that detects only extracellular dSulf1. We found that the amount of extracellular dSulf1 DDKD-SGW protein was significantly increased compared to that of dSulf1 protein. There were 1.3- to 1.4-fold increases in both the GFP-positive posterior and GFP-negative anterior areas (Fig. 4E **and fig. S19A**). As observed in the cultured cells described above, CS glycan addition also promotes the extracellular secretion of modified Sulf proteins in vivo. Next, we examined whether CS modification could alter the molecular diffusion and gradient of the extracellular dSulf1. The distance that dSulf1 extends from the boundary of the GFP-positive region in the wing pouch was measured. No significant difference in the spread distance of dSulf1 proteins between hh>dSulf1 and hh>dSulf1 DDKD-SGW was observed (Fig. 4E). The extracellular staining assay also exhibited a comparable gradient pattern in both hh>dSulf1 and hh>dSulf1 DDKD-SGW (**fig. S19B**). dSulf1 is known to negatively regulate cell proliferation in the wing disc by reducing Wg and BMP signaling through its endoglucosamine-6-sulfatase activity directed against HSPG^35, 36^. The size of the posterior compartment is inversely proportional to the activity of dSulf1. The hh>dSulf1 DDKD-SGW strain showed greater negative regulation of cell proliferation in the posterior compartment than the hh>dSulf1 strain (Fig. 4F). These results strongly support the idea that the CS sequon indeed functions in vivo and that CS modification selectively increases the enzymatic activity without affecting extracellular diffusion of the secreted Sulf proteins. Through protein engineering utilizing the discovered CS sequon, we identified a novel functional regulatory mechanism for CS addition in the SULFs and demonstrated in vitro and in vivo that the addition of CS increases the amounts of modified proteins in the extracellular space and enhances their enzymatic activity.

Finally, our results highlight additional considerations. First, larger protein domains appear to determine whether the CS sequon is functionally utilized. In the case of the SULFs, this domain is the intrinsically disordered domain with a random coil structure. When targeting extracellular proteins other than SULFs for CS addition, these structural considerations may be important. Second, we did not observe the biosynthesis of new HS in our variants. This result suggests that another sequence for a functional HS sequon may exist. In this sequence, EXTL3 selectively specifies HS biosynthesis^37^. Identifying this sequence is a future challenge and would facilitate the elucidation of detailed mechanisms of post-translational CS and HS modifications using the CS sequon^21, 22, 29, 37, 38, 39, 40, 41, 42, 43^ (**fig. S20A**). Third, the potential involvement of the discovered CS sequon in the formation of enzyme complexes involved in CS elongation is also a future research topic^44^. Perhaps the length of CS can be regulated through molecular recognition of the modified protein by the enzymes responsible for CS elongation. This process may involve functional domains other than the CS-modified Ser residue within the core protein and a carbohydrate linkage with specific structure, such as phosphorylation, as seen in the matriglycan mechanism^45, 46, 47^. Interestingly, BioGRID data showed that CSGALNACT2 interacts with SULF2, but not with SULF1 (https://thebiogrid.org)^48^. Molecular recognition of CSGALNACT2 may influence CS biosynthesis for SULF2^49^.

In summary, we successfully identified the 16 amino acid sequence of a functional sequon, EDQDDKDGGDFSGWGG, that initiates the tetrasaccharide linkage biosynthesis and polymerization of the CS chain. Moreover, this study paves the way for designing proteins with intentionally controlled CS modifications using the CS sequon. The mechanism proposed by us, whereby CS polymerization occurs near the core protein, will be further verified in future work (**fig. S20B**). Our findings open up new avenues for studying CS-regulating neuronal plasticity in the brain^50^ by enabling the targeted delivery of engineered CS peptides and proteins to specific locations in the desired amounts.

## Methods:

### Reagents and antibodies

Materials were obtained from the following sources: Peptide-N-glycosidase F (PNGase F; P0704S, *F. meningosepticum*) was purchased from New England Biolabs (Ipswich, MA); HEK293 (CRL-1573), CHO (CCL-61), and COS-7 (CRL-1651) cells were from ATCC (Manassas, VA); The mouse anti-SULF2 antibody 8G1 (MABC586)^30^, mouse anti-chondroitin-4-sulfate stub antibody BE123 (MAB2030), mouse anti-ß actin (A2228), rat anti-HA antibody (11867423001), heparinases I (H2519), II (H6512), and III (H8891) (*F. heparinum*), and the cOmplete^™^ protease inhibitor cocktail (11836170001) were purchased from Sigma-Aldrich (St. Louis, MO); The mouse anti-His-tag antibody was purchased from MBL Life Science (D291–3, Tokyo, Japan); The rabbit anti-VSV-G antibody was obtained from Bethyl Laboratories (A190–131A, Montgomery, TX); The horseradish peroxidase (HRP)-conjugated goat anti-mouse IgG2a (115-035-206), HRP-goat anti-mouse IgG1 (115-035-205), HRP-goat anti-mouse IgM μ (115-035-075), HRP-goat anti-rabbit IgG (H+L) (111-035-144), and the alkaline phosphatase (AP)-conjugated anti-rabbit IgG (H+L) antibody (111-055-045), were obtained from Jackson ImmunoResearch Laboratories (West Grove, PA); The HA-Tag monoclonal antibody (26183), Alexa Fluor 568-conjugated goat anti-rat IgG (H+L) antibody (A-11077), ECL Western Blotting Substrate (32209), PNPP substrate (37621), and Protein G- (88847) and HisPur^™^ Ni-NTA-magnetic beads (88832) were purchased from Thermo Fisher Scientific (Waltham, MA); Protease-free Chondroitinase ABC (ChABC) (*P. vulgaris*) was obtained from Seikagaku Corporation (100332, Tokyo, Japan); The anti-chondroitin sulfate A antibody LY111 was purchased from Tokyo Chemical Industry (A3143, Tokyo, Japan). VSV-G-tagged RB4CD12 anti-heparan sulfate S-domains was previously described^33^. The anti-SULF1 antibody 4-C25^31^ and Drosophila Dmel2 cells were described previously^36^.

### Mutagenesis

Expression plasmids of the human SULF2- and SULF1-mutated variants were generated using the Q5^®^ Site-Directed Mutagenesis Kit (New England Biolabs) and the pcDNA3.1/Myc-His(−)HSulf2 and HSulf1 plasmids^1^, respectively. The primers used to construct each plasmid are shown in **table S3**. dSulf1 and its mutated variants were generated from pAW dSulf1^36^.

### Cell culture and SULF expression

HEK293 and CHO cells were grown in Dulbecco’s modified Eagle’s medium (DMEM) high glucose (Lonza, Basel, Switzerland)/10% FBS, Ham’s F12 (Lonza)/10% FBS, and DMEM low glucose/10% FBS, respectively, in 75 cm^2^ flasks at 37 °C with 5% CO_2_. Sixteen micrograms of a SULF expression plasmid were transfected using Lipofectamine 2000 according to the instructions (Thermo Fisher Scientific). After washing with PBS twice and OptiMEM (Thermo Fisher Scientific) once, the culture media were replaced with OptiMEM. DNA-Lipofectamine complexes in OptiMEM were then added to the cells. The CM were collected after 48 hs and concentrated 100-fold using an Amicon^®^ Ultra 30 kDa cut-off centrifugal filter (UFC8030, Sigma-Aldrich). The concentrated CM was used for Western blotting, SULF enzyme assay, and purification. Drosophila Dmel2 cells are grown in SF900III (Thermo Fisher Scientific) in 25 cm^2^ flasks at 25 °C. Lipofectamine 2000 was used to transfect 8 μg of pAW dSulf1 or pAW mutated dSulf1 variants. The harvested cells were homogenized with RIPA buffer (150 mM NaCl, 50 mM Tris-HCl, pH7.4, 0.1% SDS, 1% Triton X-100, 0.5% sodium deoxycholate, and 1x cOmplete). Then, the lysate was centrifuged at 14,000 g for 15 min at 4 °C. The supernatant was used for Western blotting. The concentrated CM was digested with 50 mU of ChABC and/or a mixture of 1mU of heparinase I, 0.25 mU of heparinase II, and 0.1 mU of heparinase III at 37 °C for 1 h in a 50 μL reaction mix. To remove N-glycans, concentrated CM was incubated with Glycoprotein Denaturing Buffer at 100 °C for 10 min. Then, the samples were digested with PNGase F in Glyco Buffer containing 1% NP-40 at 37 °C for 1 h according to the manufacturer’s instructions.

### Immunoblot

The concentrated CMs and proteins (20 μg) were separated using 7.5% polyacrylamide gel electrophoresis, then blotted onto a polyvinylidene difluoride membrane (Thermo Fisher Scientific). The membrane was blocked with 5% skim milk in TBS containing 0.1% Tween-20 (TBS-T) for 1 h at room temperature. Then, the membrane was incubated overnight at 4 °C with the following primary antibody dilution in 5% skim milk/TBS-T: anti-His-tag (1:1000), anti-HSULF2 8G1 (1:700), anti-HSULF1 4-C25 (1:1000), anti-chondroitin-4-sulfate stub BE123 (1:500), anti-CS-A LY111 (1:1000), anti-HA-tag (1:1000), or anti-β-actin (1:1000). The membranes were washed with TBS-T and incubated with HRP-conjugated secondary antibodies for 1 h at room temperature. The bound antibodies were visualized using an ECL chemiluminescent reagent and detected with a Fusion Solo system (Vilber, Marne-la-Vallée, France). Densitometry analysis of immunoreactive bands was performed with the ImageJ software (http://imagej.nih.gov/ij/) (NIH, Bethesda, MD).

### Immunoprecipitation

The His-tagged SULF variant proteins (approximately 10 μg) in concentrated cell culture CM prepared from 20 mL of cultured cell OptiMEM medium were pretreated with 200 mU of ChABC for 2 hs at 37 °C. Then, the mixture was incubated with HisPur^™^ Ni-NTA magnetic beads for 30 min at room temperature. The beads were isolated using a DynaMag^™^−2 magnet (Thermo Fisher Scientific), then washed three times with a wash buffer containing 100 mM phosphate buffer (PB) (pH 8.0), 600 mM NaCl, 50 mM imidazole, and 0.05% Tween 20, followed by three additional washes with PBS. The bead-bound materials underwent protease treatment followed by molecular identification using LC-NSI-MS/MS.

### Nano LC-MS/MS analysis

Ni-NTA magnetic bead-bound SULF proteins were reduced, alkylated, and on-bead digested with trypsin or Lys-C. Briefly, the SULF protein-bound beads were washed with PBS to remove residual detergent from the immunoprecipitation wash buffer. The beads were then vortexed, centrifuged at 6,000 × g for 10 s, and the supernatant was removed. The beads were resuspended in 50 μL of 50 mM ammonium bicarbonate, and 12.5 μL of 25 mM DTT was added. The mixture was incubated at 50 °C for 45 min to reduce disulfide bonds on cysteine residues. This was followed by addition of 12.5 μL of 90 mM iodoacetamide for carbamidomethylation at room temperature for 45 min in the dark. Next, 1 μg of trypsin or Lys-C was added to the beads, and the digestion was carried out at 37 °C overnight. After digestion, the supernatant was recovered by centrifuging the beads at 6,000 × g for 20 s. Fifty microliters of 50 mM ammonium bicarbonate were added to the beads, briefly vortexed, and the beads were centrifuged again at 6,000 × g for 20 s. The supernatants were combined and dried. Nano LC–MS/MS analysis^18, 19^ was performed on an Orbitrap Eclipse Tribrid Mass Spectrometer with an EASY nanospray source and Ultimate3000 autosampler LC system (Thermo Fisher Scientific). Approximately 0.5–1 μg of digest was separated on a nano-C18 column (Acclaim pepMap RSLC, 75 μm × 150 mm, C18, 2 μm particle size) with a 180-min gradient of increasing mobile phase B (80% acetonitrile, 0.1% formic acid in distilled H_2_O) maintained at 60 °C at a constant flow rate of 300 nL/min routed directly into the mass spectrometer. Full MS spectra were acquired in the Orbitrap at 60,000 resolution (m/z 200), followed by data-dependent HCD MS/MS acquisition (top-speed scan, 3 s).

The resulting data were annotated by manual interpretation following initial processing using Byonic software (version 4.1.10, Protein Metrics). Byonic parameters were set to allow a 20 ppm tolerance for both precursor ion monoisotopic mass and fragment ions. Searches were performed against the SULF protein sequences with differential modifications including carbamidomethylation of cysteine (+57.02146 Da) and oxidation of methionine (+15.9949 Da). In addition, human/mammalian N-glycosylation, O-glycosylation, and a series of CS-core mono- and oligosaccharide compositions were included to analyze post-translational glycan modifications on the proteins. All CS glycopeptide assignments were subsequently manually validated by direct inspection of MS/MS spectra. Identification relied on diagnostic fragment ions derived from the Δ^4^-unsaturated uronic acid produced by ChABC digestion, generating the characteristic UHexA–HexNAc fragment ion at m/z 362, together with peptide backbone fragment ions confirming site localization. A key analytical challenge was the presence of glycopeptide species containing either a single phosphate on xylose (ΔM = 79.9663 Da) or a single sulfate on GalNAc (ΔM = 79.9568 Da). These modifications differ by only 0.0095 Da and are indistinguishable at the precursor mass level, even at high Orbitrap resolution. Assignments did not rely on precursor mass accuracy but instead on diagnostic MS/MS fragmentation behavior. Xylosyl-phosphorylated species generated fragment ions corresponding to the peptide bearing xylose and phosphate, confirming phosphorylation at the xylose residue. GalNAc-sulfated species produced characteristic neutral losses and fragment ions corresponding to the sulfated UHexA–HexNAc disaccharide at m/z 442. These species frequently co-eluted partially, with the xylosyl-phosphorylated form eluting slightly earlier than the GalNAc-sulfated form. Final assignments were manually curated based on these diagnostic features. Relative quantification was performed by integrating extracted ion chromatograms across all observed charge states within the chromatographic elution window. For partially co-eluting phosphate and sulfate isomers, relative abundance was determined from the intensities of their diagnostic MS/MS fragment ions in averaged spectra rather than from precursor signal alone. All CS core glycopeptide structures, modification states, and site localization were determined through combined software-assisted identification and manual spectral interpretation. This approach was necessary due to the structural complexity of the glycopeptides and the near-isobaric nature of phosphate and sulfate modifications (**table S4, S5 and S6**).

### Peptide synthesis

The nine different peptide sequence of SULF2, SULF2 variants, and SULF1 are shown (see Fig. 3 **and fig S9**). To ensure the data is more relevant to the secondary structure of intact SULF, the peptides were designed to be more than 18 amino acids long. For SULF2 and its variants, we synthesized C-terminal extended forms with Leu-Pro-Asp. For SULF1, we synthesized a C-terminal extended form with Leu-Ala-Asp. All were generated as hydrochloric salts (GenScript, Tokyo, Japan). The purity and molecular weight (MW) of each peptide were identified using reversed-phase LC and electrospray ionization MS, respectively (see **data S1**). The results are as follows: SULF2 pep (93.6%, 1925.4 for theoretical MW: 1924.85); SULF2 AA pep (93.5%, 1922.0 for theoretical MW: 1922.88); SULF2 (SG)_3_ pep (92.1%, 2357.1 for theoretical MW: 2357.24); SULF2 DLQA pep (93.2%, 1816.6 for theoretical MW: 1816.69); SULF2 SSGG pep (90.0%, 2091.9 for theoretical MW: 2092.13); SULF2 W (T585W) pep (94.2%, 2009.6 for theoretical MW: 2009.96); SULF2 K577A pep (90.2%, 1867.6 for theoretical MW: 1867.75); SULF2 F582A pep (92.2%, 1848.4 for theoretical MW: 1848.75); SULF1 pep (96.3%, 1958.1 for theoretical MW: 1958.13). Stock solutions of the peptides were prepared in water. Absorbance of Trp/Phe at 280 nm (5500 M^−1^ cm^−1^) was measured to determine concentrations of some peptide solutions using a NanoDrop One (Thermo Fisher Scientific).

### Circular dichroism analysis

The secondary structure of the CS sequon was evaluated by circular dichroism (CD) spectroscopy using the synthesized peptides. Peptide solutions (200 μg/mL) in 10 mM Tris buffer containing 150 mM NaCl (pH 7.4) were subjected to CD measurements. Far-UV CD spectra were recorded from 190 to 260 nm at 25 °C using a J-1100 CD spectrometer (JASCO, Tokyo, Japan) with a 1-mm path length quartz cuvette as described previously^28^. The spectrum of the corresponding buffer was subtracted from each peptide spectrum for baseline correction.

### Xylosyltransferase assay

Expression plasmids, p3xFLAG-CMV8, containing truncated form of human XYLT2 and XYLT1 were transiently transfected into COS-7 cells by Lipofectamine 3000. Cells were harvested after 48 hs and then the CM were collected. XYLT2 and XYLT1 proteins were purified using anti-FLAG tag affinity beads. Purified XYLT proteins were quantified by fluorescence Western blotting using an anti-FLAG antibody and an anti-mouse IgG IRDye 680 (Li-Cor Biotechnology, Lincoln NE). The standard reaction mixture contained 25 mM MES-NaOH, pH 6.5, 25 mM KCl, 5 mM KF, 5 mM MnCl_2_, 5 mM MgCl_2_, 0.1 mM uridine diphosphate (UDP)-xylose, 2.5 nmol of synthesized peptides, and 10 μl of the purified XYLT protein-bound beads (50 ng of XYLT2, 160 ng of XYLT1), in a final volume of 50 μl. After incubating at 37 °C for 1 h, the amount of UDP released as a product of xylosyltransferase in the reaction mixture was quantified using a UDP-Glo^™^ glycosyltransferase bioluminescenct assay kit (V6963, Promega, Madison WI). The microplate luminometer EnSpire (PerkinElmer, Shelton CT) was used to detect the luminescence signals. When using a partially purified XYLT2 and XYLT1, control CM were prepared from COS-7 cells that were transfected with an empty 3xFLAG-CMV8 vector. The UDP detection signals in this control were calculated as blanks for all detected values. The activity of mono-xylosylation towards the serine in the synthesized peptides was determined by matrix-assisted laser desorption/ionization-time of flight (MALDI-TOF)-MS (Autoflex speed, Bruker, Billerica MA).

### ELISA of endoglucosamine-6-sulfatase assay

Eight micrograms of SULF proteins were purified from concentrated CM of cultured cells using HisPur^™^ Ni-NTA magnetic beads. The bead-bound proteins were then resuspended in 200 μl of 50 mM HEPES buffer, pH 7.5. An Immulon^®^ 2 HB 96-well microtiter plate was coated with 50 ng of heparin-conjugated bovine serum albumin (BSA) per well overnight^33^. The wells were blocked with 200 μl of 3% BSA for 1.5 h. Then, 100 μl of the reaction mix (4 μl of the resuspended bead solution containing 50 mM HEPES pH 8.0, 10 mM MgCl_2_, 250 mM imidazole) was applied to each well. The plate was incubated for 1 h at 37 °C. A mixture of heparinases I, II, and III was used as a control. Then, the wells were incubated with 100 μl of VSV-G-tagged RB4CD12 anti-heparan sulfate S-domain (1:800) for 1 h at room temperature. Next, 100 μl of rabbit anti-VSV-G antibody (1:1000) was added and incubated for 45 min. Finally, 100 μl of AP-conjugated anti-rabbit IgG antibody (1:2000) was added and incubated for 30 min. Between each step, the wells were washed with PBS-T three times. The PNPP substrate in a diethanolamide buffer solution (100 μl/well) was then added, and the OD at 405 nm was read over time for 30 min using a CLARIOstar Plus (BMG LABTECH, Champigny-sur-Marne, France). Reduced RB4CD12 signals were calculated as endoglucosamine-6-sulfatase activity as previously described^33^.

### Quantitative real-time PCR

The total RNA was extracted from cultured cells using the TRIzol reagent (Thermo Fisher Scientific). DNase I-treated RNA (5 μg) was subjected to reverse transcription in 100 μl of reaction buffer with RNase inhibitor (Sigma-Aldrich), random primers, SuperScript II Reverse Transcriptase (Thermo Fisher Scientific), and a mixture of dNTPs (Sigma-Aldrich). Quantitative real-time (qRT) PCR was performed using SYBR qPCR Mix (Toyobo, Osaka, Japan) and an Mx3000P Real-Time QPCR System (Agilent Technologies, Santa Clara, CA). The following procedure was used: 1 cycle of 95 °C for 10 s, and 40 cycles of 95 °C for 5 s and 60 °C for 30 s. The relative expression levels for each mRNA were normalized to the level of glyceraldehyde 3-phosphate dehydrogenase (GAPDH) mRNA, calculated using the 2^−ΔΔCt^ method. An appropriate control was set as the calibrator. Triplicate real-time PCR reactions were used for analysis. The primer sequences were as follows: 5′-GGCATTCCTATCCAAGAACC-3′ (forward) and 5′-CCCTGTTTCTTGATGAACCTG-3′ (reverse) for XYLT2, 5′-GTGCTAAGTCCAAGCACTG-3′ (forward) and 5′-TGTTGGCTTTACCCTCGAG-3′ (reverse) for XYLT1, and 5′-TCAAGATCATCAGCAATGCC-3′ (forward) and 5′-CGATACCAAAGTTGTCATGGA-3′ (reverse) for GAPDH.

### Drosophila strains

The following fly strains were used in this study: Oregon-R, w^1118^ (Bloomington Drosophila Stock Center [BDSC] #5905), UAS-GFP (BDSC #1521), hedgehog (hh)-GAL4; and UAS-dSulf1 (UAS-Sulf1-HA)^35^. UAS-dSulf1 DDKD-SGW-1er construct was created by cloning the dSulf1 DDKD-SGW-1er cDNA into the pUASg.attB vector. Transgenic strains bearing this construct were created by integrating the plasmid DNA into the Basler ZH line 68E using C31, as was done for the UAS-Sulf1-HA construct^35^. Flies were raised on a standard cornmeal fly medium at 25°C.

### Immunohistochemistry

Larval wing discs were dissected from third-instar wandering larvae in PBS and subsequently fixed in 3.7% formaldehyde in PBS for 40 min at room temperature. After three 10-min washes with PBST (PBS containing 0.1% Triton X-100), the samples were incubated in the rat HA-Tag monoclonal antibody (1:100) overnight at 4°C. After three 10-min washes with PBST, the samples were incubated with Alexa Fluor 568–conjugated secondary antibody (1:100) for 2 hs at room temperature. After three 10-min washes with PBST, the samples were stained with 1 μg/ml DAPI (4’6-diamidino-2-phenylindole) (62248, Thermo Fisher Scientific) and subsequently mounted in Vectashield Antifade Mounting Medium (H-1000, Vector Laboratories, Burlingame, CA). Extracellular labeling of dSulf1-HA and dSulf1 DDKD-SGW-HA was performed as previously described^35, 36^. Third instar larvae were dissected in cold M3 medium, and tissues were incubated with an anti-HA antibody in solution (1:20 dilution in M3 medium) on ice for 1 h. After incubation, samples were washed three times with PBS and subsequently fixed in 4% formaldehyde in PBS for 20 min at room temperature. Fixed tissues were washed three times with PBS for 10 min each at room temperature. Signal amplification was performed by incubating samples in solution with a biotinylated secondary antibody (1:100) overnight at 4 °C. After incubation, samples were washed three times with PBS and then incubated with fluorophore-conjugated streptavidin (1:200) for 2 hs at room temperature. Samples were subsequently washed three 10-min washes with PBST and then stained with DAPI for 30 min. Extracellular labeling of the Wg protein was performed using the 4D4 anti-Wg antibody as previously described^36^. Images were acquired using an LSM710 confocal microscope (Carl Zeiss, Oberkochen, Germany). To quantify the staining, images were acquired under the same conditions. Fluorescence intensity was measured in a set area using Fiji (https://imagej.net/software/fiji/)^36^.

### Statistical analysis

The values were analyzed using a Student’s *t*-test or one-way ANOVA with Dunnett’s test (versus the non-mutated form) or Tukey’s test via Prism (GraphPad Software, La Jolla, CA, USA). *P*-values less than 0.05 were considered statistically significant.

## Supplementary Material

Supplementary Files

This is a list of supplementary files associated with this preprint. Click to download.

- NatChemBiolDiscoveryCSSequonUchimuraSupplementaryInformationKU1withoutTC.pdf

Figs. S1 to S20

Tables S1 to S6

Data S1

## Data Availability

All data and materials supporting the findings of this study are available from the corresponding author upon reasonable request. MS raw data have been deposited in GlycoPOST under accession number GPST000702 and are publicly available for data sharing.
